# Ethionine-mediated reduction of *S*-adenosylmethionine is responsible for the neural tube defects in the developing mouse embryo-mediated m6A modification and is involved in neural tube defects via modulating Wnt/β-catenin signaling pathway

**DOI:** 10.1186/s13072-021-00426-3

**Published:** 2021-12-04

**Authors:** Li Zhang, Rui Cao, Dandan Li, Yuqing Sun, Juan Zhang, Xiuwei Wang, Ajab Khan, Zhizhen Liu, Bo Niu, Jun Xu, Jun Xie

**Affiliations:** 1 Department of Biochemistry and Molecular Biology, Shanxi Medical University, Taiyuan, 030001 Shanxi China; 2 Department of Hepatobiliary and Pancreatic Surgery, First Clinical Medical College, Shanxi Medical University, Taiyuan, 030001 Shanxi China; 3 Institute of Liver Diseases and Organ Transplantation, Shanxi Medical University, Taiyuan, 030001 Shanxi China; 4 Biology Institute of Shanxi, Taiyuan, 030001 Shanxi China; 5 Beijing Municipal Key Laboratory of Child Development and Nutriomics, Capital Institute of Pediatrics, Beijing, China

**Keywords:** Neural tube defects, M6A modification, METTL3, *S*-Adenosylmethionine, Ethionine, Wnt/β-catenin signaling pathway

## Abstract

Neural tube defects (NTDs) remain one of the most life-threatening birth defects affecting infants. Most patients with NTDs eventually develop lifelong disability, which cause significant morbidity and mortality and seriously reduce the quality of life. Our previous study has found that ethionine inhibits cell viability by disrupting the balance between proliferation and apoptosis, and preventing neural stem cells from differentiating into neurons and astrocytes. However, how ethionine participates in the pathogenesis of neural tube development through N6-methyladenosine (m6A) modification remains unknown. This study aims to investigate METTL3- and ALKBH5-mediated m6A modification function and mechanism in NTDs. Herein, our results demonstrate that SAM play not only a compensatory role, it also leads to changes of m6A modification in neural tube development and regulation. Additionally, these data implicate that METTL3 is enriched in HT-22 cells, and METTL3 knockdown reduces cell proliferation and increases apoptosis through suppressing Wnt/β-catenin signaling pathway. Significantly, overexpression of ALKBH5 can only inhibit cell proliferation, but cannot promote cell apoptosis. This research reveals an important role of SAM in development of NTDs, providing a good theoretical basis for further research on NTDs. This finding represents a novel epigenetic mechanism underlying that the m6A modification has profound and lasting implications for neural tube development.

## Introduction

Neural tube defects (NTDs) are increasingly prevalent which mainly include anencephaly, spina bifida and encephalocele. The incidence of NTDs is 0.5–2/1000, especially in Shanxi Province of China, where the incidence is as high as 13.9/1000 [1]. The pathogenesis of NTDs involves both genetic and environmental factors. Many results suggest that folate deficiency is a risk factor for NTDs [2–4]. At present, folic acid has become a new treatment for NTDs, however, it cannot prevent all types of NTDs [5]. Therefore, there is an urgent need to develop a new intervention strategy to prevent and treat NTDs. Recently, it has been reported that exogenous folic acid may prevent NTDs by regulating epigenetic modification and/or cell proliferation [6–8]. However, the research on its mechanisms was not deep enough.

Ethionine is a natural compound, and it is an *S*-ethyl analog of methionine with a small change in structure [9, 10]. It competes with methionine in combination with methyladenosyltransferase (MAT), leading to a reduction in *S*-adenosylmethionine (SAM) during the synthesis of DNAs, RNAs and proteins [11]. Some studies have found ethionine can cause NTDs by blocking the methionine cycle [12, 13]. However, research on ethionine-induced NTDs is currently limited. Previous studies have revealed that ethionine can induce NTDs, leading to a one-carbon unit metabolic disorder, reduced SAM level and SAM/SAH ratio. However, the specific pathogenesis of NTDs caused by epigenetic modification due to SAM decline remains elusive.

More and more studies have shown that the epigenetic changes caused by folate deficiency are closely related to the occurrence of NTDs, and some novel hypotheses have been proposed. It has been found that DNA hypomethylation leads to the occurrence of NTDs [14] and folate deficiency can change histone H2A monoubiquitination, affecting neural tube closure [15]. Homocysteine (Hcy) regulates the occurrence of NTDs by upregulating H3K79Hcy [16]. Although these studies have revealed the mechanism of NTDs, no effective interventions have been proposed. M6A is the most common and highly conserved RNA modification in eukaryotic cells, and it affects the stability, alternative splicing, nuclear export and translation efficiency of mRNA metabolism [17]. Increasing evidences indicate that m6A modifications are present in many physiopathological processes of cell apoptosis and survival [18]. In recent years, m6A modification has attracted great interests in the field of embryonic development. It is known that METTL3 is one of the key methyltransferases in m6A modification [17]. Several studies have recently confirmed that METTL3-mediated m6A modification is essential in mammalian embryonic development [19, 20]. Research reports METTL3 is essential for early embryogenesis before or during gastrulation in mice and zebrafish [21]. However, the complex mechanism of epigenetics of NTDs has not yet to be determined.

Neural tube is regulated by precise programming among various developmental regulation-related signaling pathways in the closure process [22, 23]. Studies have shown that Wnt/β-catenin signaling pathway is one of key signaling pathways, which affects the development of the neural layer and the failure of neural tube closure [24]. Wnt/β-catenin signaling regulates embryo development in all aspects of embryonic pattern and cell size including cell movement and tissue polarity [25, 26]. Wnt/β-catenin signaling has been shown to play an important role in maintaining self-renewal and regulating NSC differentiation [27]. Previous research has also found that folic acid supplementation protected against PM2.5 cardiac development toxicity by regulating Wnt/β-catenin signal pathways [28]. Our previous study finds that low folate promotes Wnt/β-catenin signaling by activating Gcm1, and ultimately lead to NTDs [4]. However, the regulatory mechanism by which the Wnt/β-catenin signaling pathway affects neural tube closure is unclear.

In this study, we determine that m6A modification is closely related to NTDs and that METTL3 defect leads to reduced proliferation in HT-22 cells and result in excessive cell apoptosis via suppressing Wnt/β-catenin signaling pathway. Mechanically, we propose that ethionine inhibits the Wnt/β-catenin signaling pathway by reducing m6A modification, especially the METTL3- and ALKBH5-mediated m6A modification, which causes the imbalance of cell proliferation and apoptosis, and participates in the occurrence of NTDs. Our data provide the theoretical evidence of the NTDs arising from failure for m6A modifications.

## Materials and methods

### Animals

All C57BL/6 mice (9–10 weeks, 19–25 g) procedures were approved from the Animal Laboratory Center of Shanxi Medical University, Taiyuan, People's Republic of China. The procedure was in accordance with the "Guidelines for the Use of Nursing Animals" issued by the National Institutes of Health (NIH, 8th Edition, 2011). The experimental protocol was approved by the Experimental Animal Management Committee of Shanxi Medical University. The mice were maintained on a 12-h light/dark cycle (lights on from 8:00 a.m. to 8:00 p.m.). On day 7.5 of pregnancy (E7.5), ethionine (Sigma-Aldrich, USA) was intraperitoneally injected only once at a dose of 500 mg/kg to establish the NTDs embryo model. And SAM (MedChemExpress, USA) was intraperitoneally injected only once at a dose of 30 mg/kg. The same dose was intraperitoneally injected to the pregnant mice for control group.

### Cell culture and treatments

Immortalized hippocampal neuron cell (HT-22), maintained in in DMEM (Hyclone, Logan, UT, USA), supplemented with 10% fetal bovine serum (FBS, Gibco, USA). All cells were incubated in atmosphere with 5% CO_2_ at 37 °C. The cells were treated with 20 mmol/L ethionine, 2 mmol/L SAM for 48 h. The siRNA-METTL3 plasmid was synthesized by Sangon Biotech (Shanghai, China). The METTL3 plasmid was synthesized by Gene Pharma (Shanghai, China), and transfected into the cells using Lipofectamine RNAiMax (Santa Cruz) according to the manufacturer’s instruction. The sequence of SiMettl3 was forward: 5′-GCUGCACUUCAGACGAAUUTT-3′, reversed: 5′-AAUUCGUCUGAAGUGCAGCTT-3′, and that of the negative control (siCon) was forward: 5′-UUCUCCGAACGUGUCACGUTT-3′, reversed: 5′-ACGUGACACGUUCGGAGAATT-3′. The sequence of siALKBH5 was forward: 5′-GGAUAUGCUGCUGAUGAAATT-3′, reversed: 5′-UUUCAUCAGCAGCAUAUCCTT-3′, and that of the negative control (siCon) was forward: 5′-UUCUCCGAACGUGUCACGUTT-3′, reversed: 5′-ACGUGACACGUUCGGAGAATT-3′.

### Hematoxylin–eosin staining

The E10.5 embryos paraffin sections were deparaffinized with xylene, soaked in 100, 95, 80 and 75% ethanol for 3 min each. After washing with distilled water for 2 min, hematoxylin stain was performed for 5 min. Hydrochloric acid and ethanol were applied for 30 s, washed again 5 times under running tap water and soaked in tap water for 15 min. Post keeping in eosin solution for 2 min, normal dehydration, clearing and neutral resin sealing were carried out.

### Enzyme-linked immunosorbent assay for SAM and SAH

The concentrations of SAM and SAH in embryonic tissue were determined by enzyme-linked immunosorbent assay (ELISA; Elabscience®, Wuhan, China).

### m6A RNA methylation quantification

The m6A RNA Methylation Quantification Kit (Abcam, Massachusetts, US) uses colorimetry to quantify the m6A modification in the total RNA of the sample. Total RNA was isolated from embryonic brain tissues and its concentration were detected, negative control, positive control and sample RNA to assay wells with Binding Solution for 90 min at 37 °C according to the instructions. After the capture antibody was incubated at room temperature for 60 min, the detection antibody and enhancement solution were added and incubated for 30 min, respectively. The Developer Solution was added to each well and incubated for 10 min away from light at room temperature. When the color in positive control wells turned medium blue, added Stop Solution and detected the absorbance at 450 nm within 10 min.

### TUNEL staining assay

Apoptosis was assessed using an In Situ Cell Death Detection kit, POD (Roche, USA). The E10.5 embryos paraffin sections were removed, incubated and cells were permeated at 37 °C for 20 min. Then the TUNEL staining assay was performed as described previously [13].

### Immunofluorescence analysis

The sections were identified by the standard immunofluorescence staining. The following primary antibodies were used: rabbit anti-Cyclin D1 (1:200; ab16663; Abcam, Massachusetts, US), rabbit anti-β-catenin (1:100, Cell Signaling Technology), PCNA (1:200, Santa Cruz Biotechnology, sc-56), TCF-4 (1:100, Santa Cruz Biotechnology, sc-166699), and secondary antibodies used as Goat Anti-rabbit IgG H&L (Invitrogen, A11011). Nuclei were counterstained with DAPI (Sigma-Aldrich). Images were captured using a fluorescence microscope (Nikon, Tokyo, Japan).

### Protein isolation and Western blot

Total protein was extracted from cells or embryo brain tissues by RIPA lysis buffer (Solarbio, Beijing, China) supplemented with a protease inhibitor cocktail (Sigma-Aldrich, USA) and 10 mM PMSF (Solarbio, Beijing, China), and protein was assayed by the BCA protein assay kit (Thermo Scientific). 20 µg proteins were separated by 12% SDS-PAGE and transferred to polyvinylidene fluoride (PVDF) membranes (Millipore, Billerica, MA, USA), which were then blocked with 5% skim milk in PBST (PBS with 0.05% Tween-20) for 1 h at room temperature. The transferred proteins were reacted with primary antibody overnight at 4 °C and then labeled with secondary antibody for 1 h at room temperature. The primary antibodies used were antibody against β-catenin (1:1000, Cell Signaling Technology), TCF-4 (1:100, Santa Cruz Biotechnology, sc-166699), Axin-2 (1:1000, Abcam, ab32197), C-myc (1:1000, Abcam, ab32072), CyclinD1 (1:500, Abcam, ab16663), PCNA (1:200, Santa Cruz Biotechnology, sc-56), BCL-2 (1:1000, Abcam, ab182858), Cleaved Caspase-3 (Asp175) (1:1000, Cell Signaling Technology, 5A1E), ALKBH5 (1:500, Abcam, ab195377), METTL3 (1:700, Abcam, ab195352), rabbit anti-β-Tubulin (1:1000; Abcam, Massachusetts, US) and secondary antibodies were goat anti–rabbit IgG (1:3000; ZB-2301; ZSGB-BIO, Beijing, China), and goat anti–mouse IgG (1:3000; ZB-2301; ZSGB-BIO, Beijing, China), and β-Tubulin was used as a housekeeping control. The protein complexes were visualized using an enhanced chemiluminescent (ECL) blot detection system (ChemiDocTM Imaging Systems, BIO-RAD, USA) following the manufacture’s instruction.

### Flow cytometric analysis of cell apoptosis and cell cycle

The Annexin V-FITC/PI apoptosis detection kit was used for the apoptosis assay (KeyGEN BioTECH, Nanjing, China). The cell cycle detection kit (KeyGEN, Suzhou, China) was used for the cell cycle analysis. Then the cell apoptosis and cell cycle assay were performed as described previously [10]. Experiments were performed three times for each group. Both data analyze with FlowJo 7.6 software.

### EDU analysis of cell proliferation

Cell proliferation was also estimated using Cell-Light Edu Apollo DNA in vitro Kit (RiboBio, Guangzhou, China). Proliferative cells were visualized and imaged using a Zeiss LSM 510 META Laser Scanning Confocal Microscopy (Nikon, Tokyo, Japan). Proliferative cells were counted in different optical fields (magnification × 200) selected in a random manner and analyzed by the software of Image J.

### Bromodeoxyuridine analysis of cell proliferation

Cells were incubated in a 24-well plate, treated with SiMettl3 and SiNc for 24 h, washed 3 times with PBS for 5 min each time and placed on a shaker with slight shaking. Added 1 ml 4% polyoxymethylene at room temperature for 30 min. Then added 2 mol/L HCl in 37 ℃ for 10 min, mixed in 1 ml 0.1% Triton X-100 at room temperature for 5 min, washed 3 times with PBS for 10 min each time, used 10% goat serum to close at 37 ℃ for 60 min. Added the diluted bromodeoxyuridine antibody overnight at 4 ℃. The next day, after 2 h incubation at room temperature, washed 3 times with PBS, added the corresponding secondary antibody at 37 °C for 2 h. Washed 3 times with PBS. Then added appropriate DAPI to the wells at room temperature for 8 min. Washed 3 times with PBS for 10 min and observed under a microscope.

### AO-EB analysis of cell apoptosis

Detection of apoptosis by AO-EB double staining (Solarbio, Beijing, China) was also performed. The cells were cultured in the 24-well plate; 24 h after with ethionine and SAM treatment, the residual medium and the non-adherent cells were removed by washing with PBS and adding fresh PBS to the cells. A volume (20 ml) of working solution per milliliter of PBS was added (according to the dosage, mixing AO solution and EB solution into the working volume at a 1:1 ratio). After incubation for 3 min at room temperature, cells were observed using a fluorescence microscope (Nikon, Tokyo, Japan).

### Total RNA extraction, reverse transcription, and quantitative PCR (qRT-PCR)

The m6A methylase and demethylase expression levels were determined in E10.5 embryonic brain tissues treated with ethionine. Total RNA was extracted using Trizol method (Invitrogen, Carlsbad), and cDNA was synthesized using Revert Aid First Strand cDNA Synthesis Kit (Thermo, USA). Maxima SYBR Green/ROX qPCR Master Mix (Takara, Japan) were used for qPCR. The data were analyzed with 2^−△△^Ct method and the mRNA level was normalized with β-actin (B661302-0001, Sangon, Shanghai). Primer sequences are shown in Table 1.

**Table 1:** Primer sequences used for RT-qPCR analysis

Symbol	Forward primer (5′–3′)	Reverse primer (5′–3′)
*Mettl3*	CGCTGCCTCCGATGTTGATCTG	ACTGACCTTCTTGCTCTGCTGTTC
*Mettl14*	ACACCCCGACCTGACTTGACTG	CCCACTCCTGCCACTCCTCTG
*Kiaa1429*	CCCAGCCACAGCCGACTTTG TC	GAGGAGGTCCAGGAGGAGTTCTTG
*Alkbh5*	GGTGGTGCGTGCCTCTAATTCC	GCCTCAAACCTGCGACACTCC
*Fto*	GTCCGTCCCAGCAGCACTTTC	ACAGCACAGCAATACAAGCAGACC
*Rbm15*	TCTCCTGGCTGTACCTGGAAGTTC	TCTGAGTAGAGGCGGCAGTGTC
*Zc3h13*	TGTGCGAGATGTGCGTGATGTC	CCATAACTGCGGGCTTCTTCCTG

### Statistical analysis

Statistical analysis was performed using GraphPad Prism version 6.0 (GraphPad Software, CA) and the data were presented as mean ± SEM. Statistical differences were performed by Student’s *t*-tests for two group comparisons and one-way ANOVA for more than two group comparisons. Moreover, in one-way ANOVA analyses, LSD *t*-tests were used to estimate the significance of the results. Differences were considered statistically significant when the *p < 0.05, **p < 0.01 or ***p < 0.001.

## Results

### Ethionine induces neural tube defects by disrupting methionine cycle

**Fig. 1:**
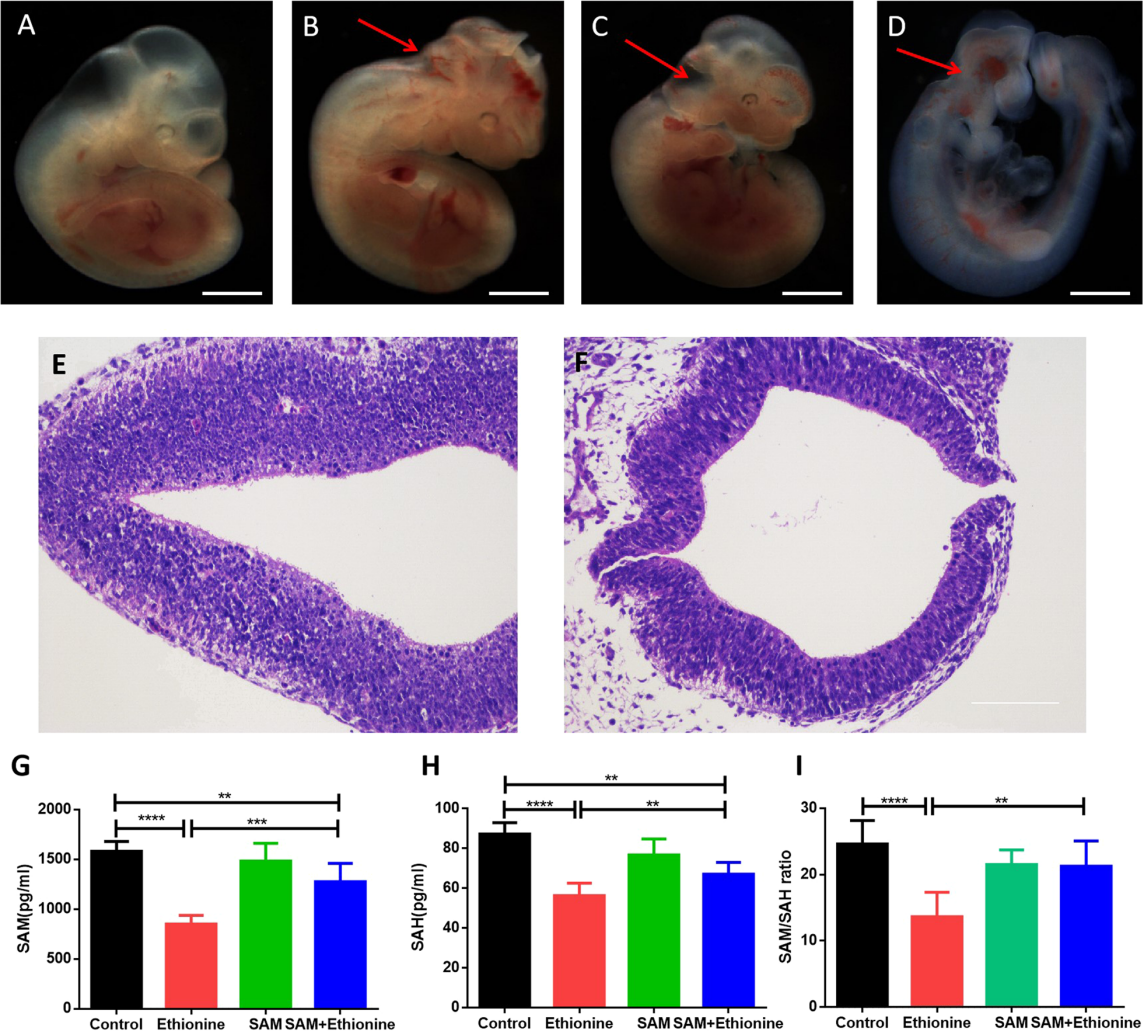
Ethionine induced neural tube defects by damaging the methionine cycle. **A**–**D** Ethionine-induced anencephaly in C57BL/6 mouse embryos at E10.5. Scale bars, 500 μm. E, **F** H&E staining results of normal and NTDs embryonic neural tube in E10.5. Scale bars, 100 μm. **G** The abundance of SAM was significantly lower in E10.5 NTDs embryos than in wild-types (*p < 0.05) SAH concentration (**H**) was elevated, and the SAM/SAH ratio (**I**) was reduced in ethionine-induced embryos compared with normal embryos (*p < 0.01, significantly differs from ethionine-induced embryos). However, the levels of SAM were increased significantly after treating with 2 mM SAM. **H** SAH and the SAM/SAH ratio (**I**) was reduced in ethionine-induced mouse embryos compared with normal embryos (**p < 0.01), which is significantly different from control. Similarly, after giving 2 mM SAM compensation, the levels of SAH and SAM/SAH ratio were obviously increased (*p < 0.05). *n* = 8 samples per genotype for each tissue

Ethionine is a natural compound, which is an *S*-ethyl analog of methionine [11]. In previous studies, we proved that 500 mg/kg of ethionine caused the highest incidence of NTDs (54.8%) [13]. Based on these theoretical foundations, ethionine and SAM were intraperitoneally injected to establish the NTDs embryo model in C57BL/6 mouse on E7.5. Results found that the incidence of NTDs was 49.3%. The incidence of NTDs was 25.6% in ethionine and SAM treatment group (Table 2), suggesting that ethionine caused NTDs via reducing the metabolic level of SAM. We found that a full appearance of the structural characteristics was observed in the control embryos (Fig. 1A). Ethionine-treated embryos displayed an obvious growth retardation and malformations along with a small and hypoplastic brain vesicle by stereomicroscope (Fig. 1B–D). Consistent with this finding, we found that the neural tube failed in the back brain region of NTD embryos compared with normal embryos by hematoxylin–eosin staining (Fig. 1E, F). Simultaneously, we also extracted the pregnant mice embryonic tissue, and found a reduced abundance of SAM in mouse embryonic tissue (Fig. 1G). Moreover, SAH abundance was significantly altered in NTDs embryonic tissue compared with control group (Fig. 1H) with a consequent reduction in the SAM/SAH ratio (Fig. 1I). In contrast, the levels of SAM and SAM/SAH ratio were increased significantly in ethionine and SAM treatment group (Fig. 1G, I). Results also showed that neural tube closure was perturbed in ethionine-induced mouse embryonic tissue. These findings suggested that ethionine induced NTDs by blocking the generation of SAM.

**Table 2:** Embryonic phenotypes of mice treated with ethionine and SAM

	Pregnant mice (*n*)	Embryos (*n*)	Normal *n* (%)	Resorption *n* (%)	Growth retardation *n* (%)	NTDs *n* (%)	Other malformation *n* (%)
SAM	36	223	207 (93.0)	6 (2.7)	10 (4.3)	0 (0)	0 (0)
Ethionine	38	261	51 (19.5)	31 (11.8)	36 (13.7)	128 (49.3)	11^a^4^b^ (5.7)
Ethionine + SAM	37	246	142 (58.1)	21 (8.1)	15 (6.1)	63 (25.6)	3^a^2^b^ (2.1)

^a^Craniofacial malformation

^b^Polydactyly

### Ethionine inhibits cell proliferation in E10.5 embryos and HT-22 cells

**Fig. 2:**
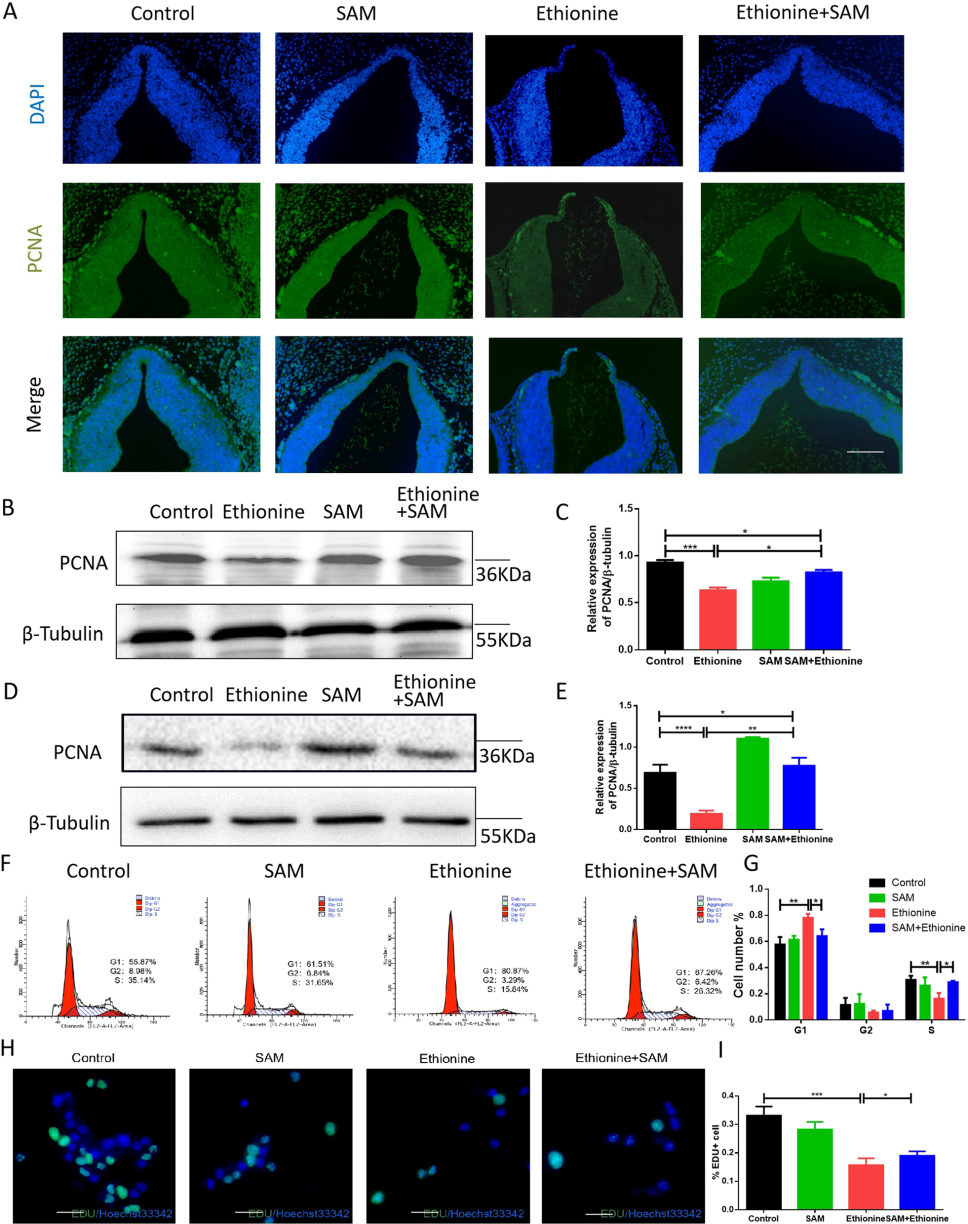
Ethionine inhibited cell proliferation in E10.5 embryos and HT-22 cells. **A** PCNA-positive cell (green) of E10.5 NTDs embryos and normal embryos by immunofluorescence. Cell nuclei were stained with DAPI (blue). The percentage of PCNA-positive cells in each region is shown. Scale bars, 500 μm. **B**, **C** Western blot detected the expression profiles of PCNA in embryonic tissue treated with ethionine and SAM. The bar graph shows the relative band intensity of PCNA. β-Tubulin levels were also evaluated to confirm equal loading (*n* = 3). ***p < 0.001 compared with WT; **p < 0.01 compared with ethionine+SAM. (D and E) Western blot analysis of the protein levels of PCNA in the HT-22 cells treated with ethionine and SAM. The bar graph shows the relative band intensity of PCNA. β-Tubulin was used as a loading control (*n* = 3; mean ± SEM; ***p < 0.001). **F**, **G** The cell cycle distribution in the HT-22 cells treated with ethionine and SAM was analyzed by flow cytometry. **H**, **I** The effect of SAM and ethionine on cell proliferation was detected using Cell-Light Edu Apollo DNA in vitro Kit and was quantified. Images of immunofluorescence cell staining against the EDU (shown in green), and the nuclei were counterstained using DAPI (shown in blue). Scale bar: 500 μm

To determine whether the ethionine-induced NTDs could be caused through an imbalance between cell proliferation and apoptosis, we detected protein expression level of proliferation-related indicators-PCNA using immunofluorescence. Our results showed that the expression of PCNA peaked at normal embryos compared with ethionine-induced embryos, and SAM+ethionine-induced embryos exhibited an increase in the density of PCNA-positive compared to ethionine-induced embryos (Fig. 2A). Western blot result found an obvious decreased level of PCNA in ethionine treatment group (Fig. 2B, C). On the contrary, the expression of PCNA was markedly increased in the ethionine and SAM-treated group compared with the ethionine-treated group (Fig. 2B–C), suggesting that ethionine-induced NTDs could be due to reduced proliferation or increased mitotic progression. Hereafter, to investigate the relationships between SAM and cell biological, we treated HT-22 cells with ethionine and SAM. We also used Western blot to detect the protein expression level of PCNA, an indicator of cell proliferation. Consistent with these findings, the protein levels of PCNA were found to be significantly lower in ethionine group than in control group, data also indicated a higher PCNA expression in SAM+ethionine induced HT-22 cell lines which implied the regulation relationship between ethionine and cell proliferation (Fig. 2D, E).

We further used flow cytometry to detect cell cycle, and the results showed that the intervention of ethionine caused aggregation in G1 phase, and cell decline in S phase. That is, cells accumulate in G1 phase and cannot transfer to S phase, inhibiting the cell from entering the process of mitosis. After ethionine combined with SAM treatment, cells in G1 phase decreased and S phase increased (Fig. 2F, G). Figure 2H, I shows that the ratio of EDU-positive cells had a conspicuous reduction in ethionine group compared with control group, indicating that SAM can promote the decline of cell proliferation caused by ethionine. All in all, these results support the notion that ethionine inhibits cell proliferation in E10.5 embryos and HT-22 cells.

### Ethionine induces cell apoptosis in E10.5 embryos and HT-22 cells

**Fig. 3:**
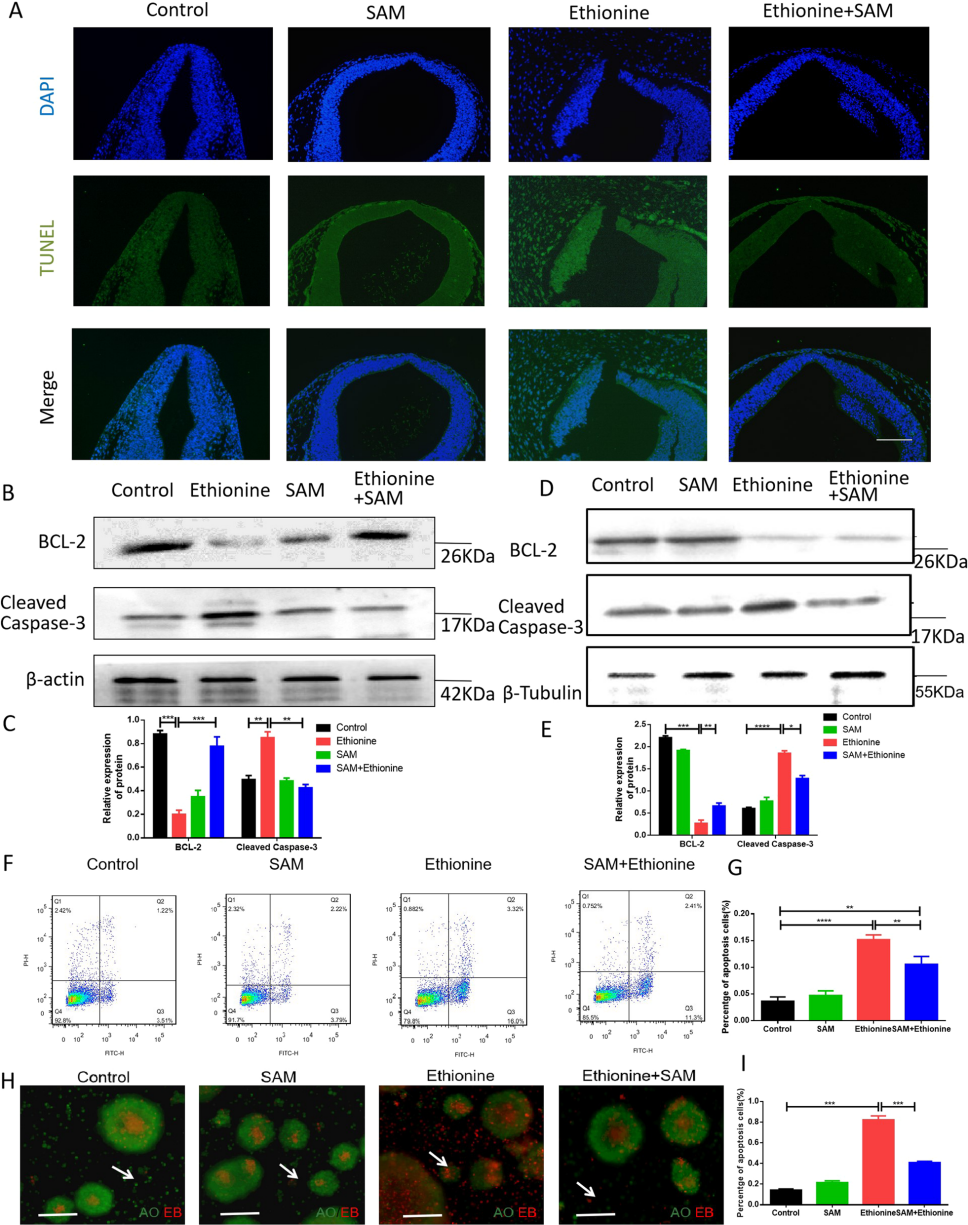
Ethionine induced excessive cell apoptosis in E10.5 embryos and HT-22 cells. **A** The cell apoptosis was examined by TUNEL assay (green) in WT, ethionine, SAM, ethionine+SAM E10.5 embryos. Cell nuclei were stained with DAPI (blue). The percentage of PCNA-positive cells in each region is shown. Scale bars, 500 μm. **B**, **C** BCL-2, Cleaved Caspase-3 protein levels in WT, ethionine, SAM, ethionine + SAM embryos brain in E10.5 were evaluated via Western blot and quantified; β-actin levels were also evaluated to confirm equal loading (*n* = 3). Each experiment was carried out in triplicates ***p < 0.01 vs. control group. (D and E) BCL-2 and Cleaved Caspase-3 in HT-22 cells treated with ethionine and SAM. β-Tubulin was used as a loading control. Bar graphs for protein abundance were quantitative data from three independent experiments. **F**, **G** The cell apoptosis in the HT-22 cells treated with ehionine and SAM was analyzed by flow cytometry. **H**, **I** Cell apoptosis was detected by AO-EB double staining and quantified. The late apoptosis cells with bright red fluorescence in ethionine-treated groups were significantly increased compared with that in the control group and ethionine+SAM group. Scale bar: 100 μm

Cell proliferation and apoptosis are equally important. To further analyze the difference in TUNEL-positive cell positioning after ethionine induced, we detected the ratio of TUNEL-positive cells on serial histological sections. There was an obvious increase in TUNEL-positive cell numbers in ethionine-induced embryos compared with normal group (Fig. 3A). Moreover, we also evaluated that there was a significant upregulation and downregulation of Cleaved Caspase-3 and BCL-2 protein, respectively, in ethionine-induced embryos compared with the normal embryos (Fig. 3B, C).

Next, we evaluated whether SAM was necessary in HT-22 cell lines for regulation of BCL-2 and Cleaved Caspase-3 by Western blot. As shown in Fig. 3D, E, a significant upregulation of Cleaved Caspase-3 proteins was detected after ethionine treatment. In contrast, the expression of the anti-apoptotic protein BCL-2 was reduced. Flow cytometry analysis found that the combined early (lower right quadrant) apoptosis rates were rise significantly in the ethionine group compared with cells in control group, and cell apoptosis rates were reduced in the ethionine+SAM group compared with ethionine group (Fig. 3F, G). This work also used AO/EB staining to detect cell apoptosis, and we found that the ethionine group can induce cell apoptosis (0.1186 ± 0.009862 vs 0.8547 ± 0.0132, *p* < 0.001), in contrast, cell apoptosis rates were reduced in the ethionine+SAM group compared with ethionine group (0.3437 ± 0.006241 vs 0.8547 ± 0.0132, *p* < 0.001) (Fig. 3H, I). These results showed that ethionine induced apoptosis in the embryos treated with ethionine. Excessive apoptosis and reduced proliferation are manifested in the ethionine-induced NTDs embryos, however, apoptosis decreased and cell proliferation increased after SAM supplementation. Thus, subsequent studies were performed in how does ethionine caused cell proliferation and apoptosis imbalance? Then we determined the causal relationship between reduced proliferation/enhanced apoptosis and NTD formation.

### Ethionine changes m6A modification through abnormal expression of m6A methylase and demethylase

**Fig. 4:**
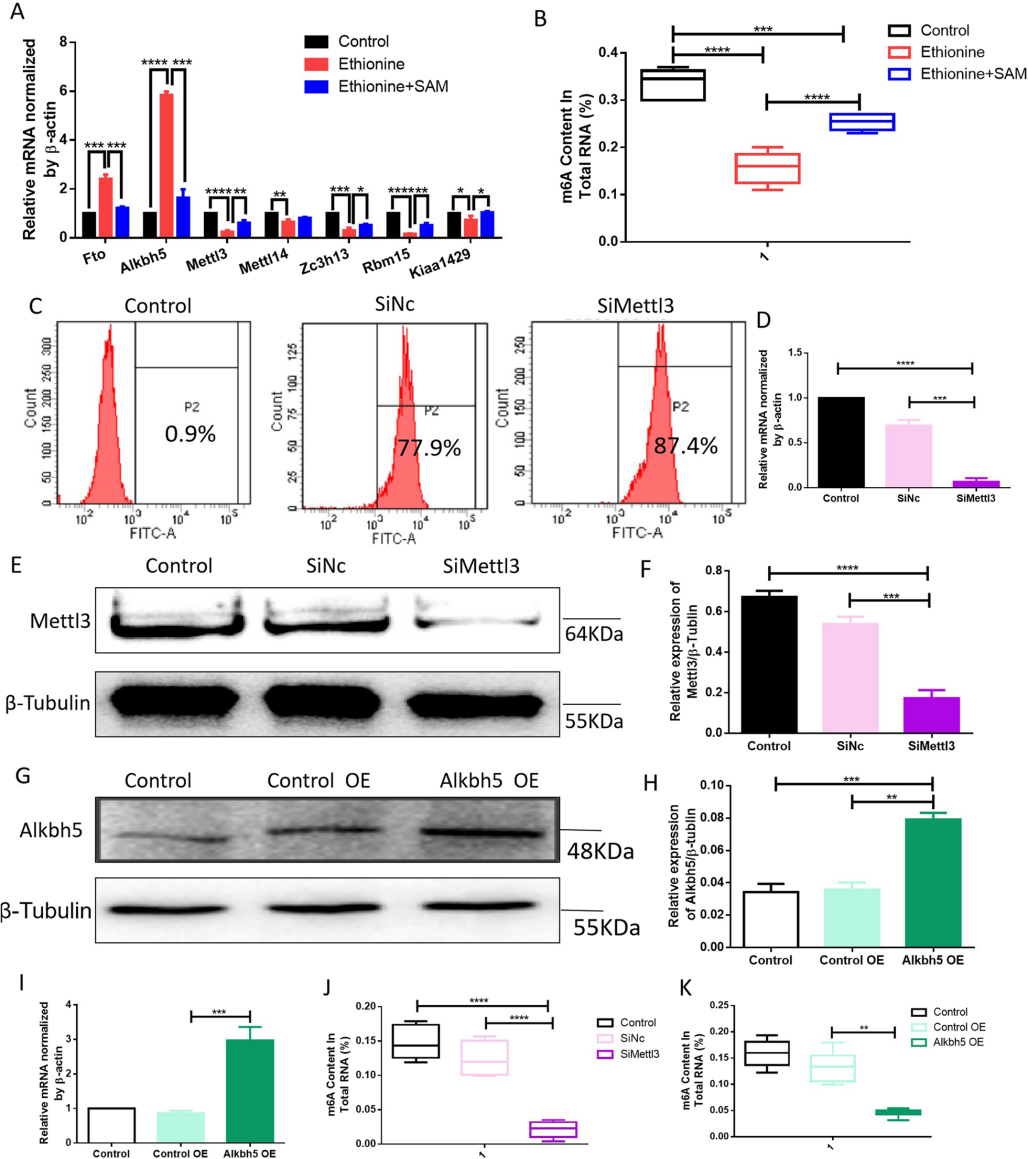
Ethionine changes m6A modification through abnormal expression of m6A methylase and demethylase. **A** RT-qPCR was used to detect mRNA level of N6-methyladenosine RNA methylase and demethylase in normal and NTDs embryo brain tissue at E10.5. *p < 0.05, **p < 0.01 and ***p < 0.001 vs control. **B** ELISA was used to detect m6A levels in normal and NTDs embryo brain tissue at E10.5. ***p < 0.001 and ****p < 0.0001 vs control. **C** Flow cytometry was used to detect the effect of SiRNA transfection, and SiNc was used as a negative control. **D** Mettl3 gene expression as assessed by RT-qPCR analysis in HT-22 cells. Mean ± SEM *p < 0.05, **p < 0.01 or ***p < 0.001 vs. The β-actin gene was used as a control. **E**, **F** Western blot analysis detected the METTL3 protein level in the SiMettl3 and SiNc groups was evaluated, and the expression of β-Tubulin was used as the loading control. **G**, **H** Western blot analysis of the protein levels of ALKBH5 in the HT-22 cells treated with control OE and Alkbh5 OE. β-Tubulin was used as a loading control. Bar graphs for protein abundance were quantitative data from three independent experiments. (I) Alkbh5 gene expression as assessed by RT-qPCR analysis in HT-22 cells treated with control OE and Alkbh5 OE. Mean ± SEM ***p < 0.001 vs. The β-actin gene was used as a control. (J) ELISA was used to detect m6A levels in SiMettl3 and SiNc cells. ****p < 0.0001 vs control. **K** ELISA was used to detect m6A levels in control OE and Alkbh5 OE cells. **p < 0.01 vs control

Firstly, we detected the expression of *Mettl3, Mettl14, Zc3h13, Rbm15, Kiaa1429, Fto* and *Alkbh5* as shown in Fig. 4A which are essential for m6A RNA modification in embryonic brain tissue. The results found that compared with the normal group, the mRNA levels of methylases *Mettl3, Mettl14, Zc3h13, Rbm15, Kiaa1429* were decreased, and the expression of demethylase *Fto, Alkbh5* were increased in NTDs group (*p* < 0.05), compared with ethionine-induced NTDs group, there had a significant rise for the mRNA levels of methylases *Mettl3, Mettl14, Zc3h13, Rbm15, Kiaa1429* were increased, and the expression of demethylase *Fto, Alkbh5* were obviously decreased in ethionine combined with SAM group. RT-qPCR analysis observed that the m6A RNA methylases were inhibited, and the m6A RNA demethylases were specifically activated (Fig. 4A). The results showed that the abnormal expression of *Mettl3* and *Alkbh5* were most obvious. Secondly, we selected m6A RNA methylation quantification kit to detect m6A RNA methylation levels in normal embryos, ethionine-induced embryos, and ethionine and SAM-treated embryos by ELISA. We found that the m6A modification was significantly reduced in the ethionine-treated group compared with the normal group; but after the SAM supplementation, the m6A modification was obviously increased, indicating that methylation reactions could potentially be compromised in ethionine-induced embryos (Fig. 4B).

To further examine whether ethionine suppressed the expression of METTL3, we synthesized SiRNA-Mettl3 plasmids and transfected them into the cells using Lipofectamine 2000. We tested the transfection efficiency by flow cytometry, and showed that the transfection rate was as high as 87.4%, indicating that both SiNc and SiMettl3 were successfully transfected into the cells (Fig. 4C). Next the expression of Mettl3 mRNA in SiRNA-METTL3-transfected cells (SiMettl3 group) was significantly decreased compared with cells transfected with SiNc-Mettl3 (SiNc group) (Fig. 4D). Western blot analysis also showed that METTL3 was knocked down in the SiMettl3 group compared to the SiNc group (Fig. 4E, F). The expression of METTL3 both at protein and mRNA levels were consistent, confirming that METTL3 was successfully knocked down in cells. Three replicate experiments were performed with three samples in each group. Simultaneously, we tested whether METTL3 knocked down would change the m6A modification. As expected, the m6A level was dramatically reduced on METTL3 knockdown (Fig. 4J). These findings indicated that SAM might activate m6A modification by deleting the *Mettl3* gene.

Figure 4A shows that the mRNA levels of *Mettl3* and *Alkbh5* were abnormal. We next examined the effect of ALKBH5-mediated m6A on the neural tube development. We synthesized a plasmid that overexpressed ALKBH5 and transfected it into the HT-22 cells using Lipofectamine 2000. We found that the expression of Alkbh5 mRNA in SiRNA-Alkbh5- transfected cells (Alkbh5 OE group) was significantly increased compared with cells transfected with SiNc-Alkbh5 (control OE group) (Fig. 4I). Western blot analysis also showed that ALKBH5 was overexpressed in the Alkbh5 OE group compared to the control OE group and control group (Fig. 4G, H), indicating that transfection of ALKBH5-expressing plasmid dramatically elevated the mRNA and protein levels of ALKBH5. We also observed that there was a significantly decreased the m6A levels in Alkbh5 OE group compared with control OE group (Fig. 4K). These data imply that ethionine may regulate neural tube development through METTL3- and ALKBH5-mediated m6A modification.

### Ethionine suppresses Wnt/β-catenin signaling pathway by deleting *Mettl3* gene and overexpressing *Alkbh5 gene*

**Fig. 5:**
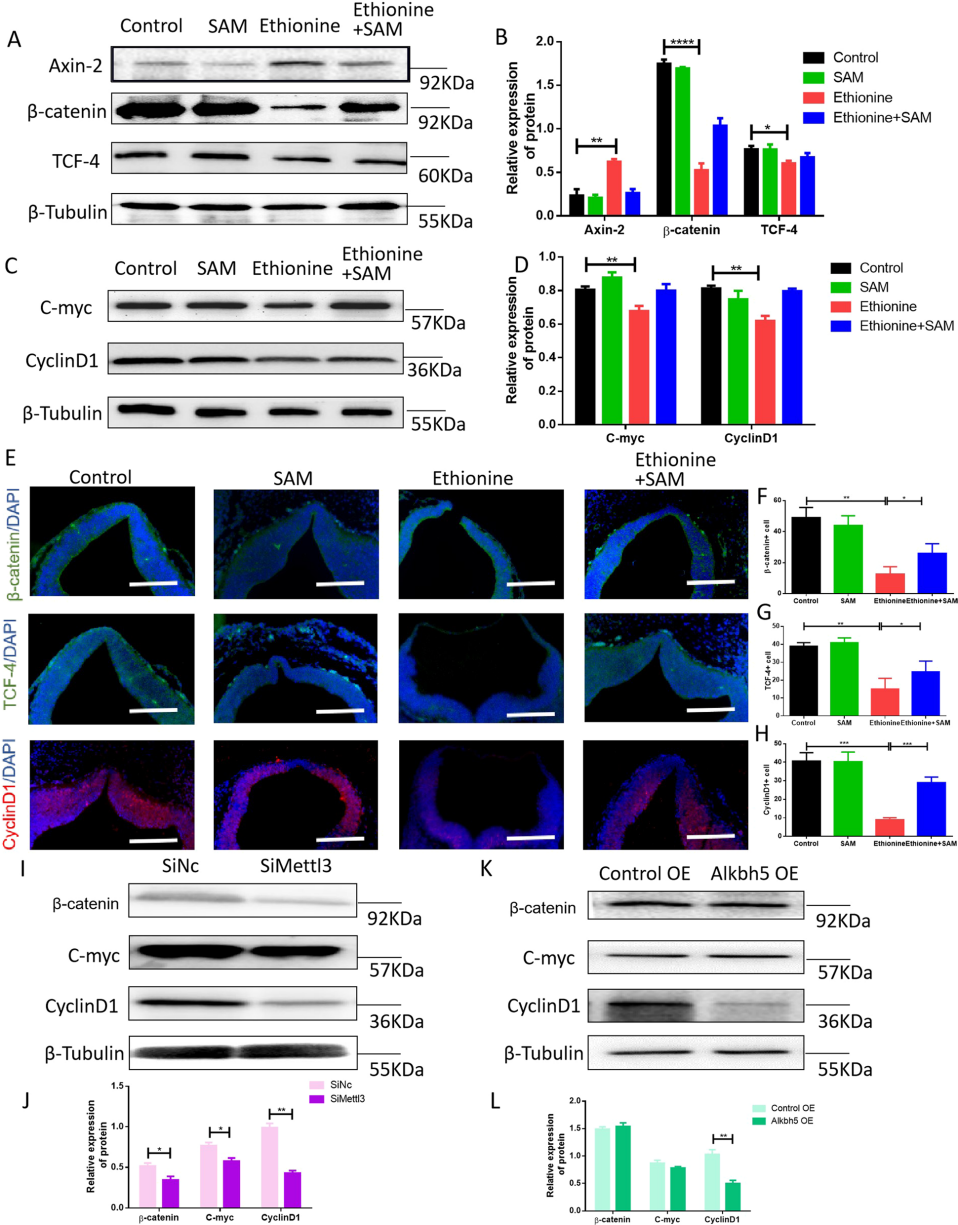
Ethionine suppresses Wnt/β-catenin signaling pathway by deleting Mettl3 gene and overexpressing Alkbh5 gene. **A**, **B** Western blot analysis of the protein levels of total β-catenin, Axin-2, and TCF-4 in E10.5 embryos. Bar graphs for protein abundance were quantitative data from three independent experiments. β-Tubulin was used as a loading control. **C**, **D** Western blot analysis of the protein levels of total CyclinD1 and C-myc in E10.5 embryos. Bar graphs for protein abundance were quantitative data from three independent experiments. β-Tubulin was used as a loading control. **E**–**H** Representative micrographs of immunofluorescent staining for β-catenin (green), TCF-4 (green), and CyclinD1 (red) to determine whether SAM activated Wnt/β-catenin signaling pathway (**E**). Nuclei stained blue with DAPI. The percentage of β-catenin-positive cells (**F**), TCF-4-positive cells (**G**), CyclinD1-positive cells (**H**) in each region is shown. **I**, **J** Western blot analysis of the protein levels of total β-catenin, CyclinD1 and C-myc in HT-22 cells treated with SiMettl3 and SiNc. Bar graphs for protein abundance were quantitative data from three independent experiments. **K**, **L** Western blot analysis of the protein levels of total β-catenin, CyclinD1 and C-myc in HT-22 cells treated with control OE and Alkbh5 OE. Bar graphs for protein abundance were quantitative data from three independent experiments. * indicated significant differences (*p* < 0.05) and ** indicated significant differences (*p* < 0.01) compared to the other groups in one-way ANOVA followed by Tukey tests

To understand whether ethionine inhibited the Wnt/β-catenin signaling pathway, and how ethionine affected neural tube development, we performed Western blot to test whether ethionine had a bad effect on Wnt/β-catenin signaling in E10.5 embryos brain tissue. Results showed that the expression level of Axin-2 was upregulated; on the contrary, the expression levels of β-catenin and TCF-4 were down-regulated in ethionine-induced embryos compared with the normal embryos (Fig. 5A, B). Subsequently, we also detected the protein expression levels of CyclinD1, C-myc, the target genes related to downstream proliferation of the Wnt/β-catenin signaling pathway, and we found that there was a significant drop in ethionine-induced embryos compared with normal embryos (Fig. 5C, D). In addition to this, we used immunofluorescence analysis to locate β-catenin, TCF-4, CyclinD1 proteins in serial paraffin sections, and the same experimental results were found as Western blot (Fig. 5E–H), which suggested that SAM supplementation successfully reversed the inhibitory effect of ethionine on Wnt/β-catenin signaling pathway (Fig. 5A–H). Together, these in vivo data support a model in which Wnt/β-catenin signaling is involved in promoting neural tube development.

Moreover, to test whether the suppression of Wnt/β-catenin signaling could be a consequence of METTL3- and ALKBH5-mediated m6A modification, we also performed Western blot analysis to detect the expression levels of Wnt/β-catenin signaling in ALKBH5 OE cells or METTL3 knockdown cells. Western blot analysis showed that the expression levels of β-catenin, CyclinD1 and C-myc were significantly down-regulated in SiMettl3 group compared with SiNc group (Fig. 5I, J). On the contrary, the protein expressions of β-catenin and C-myc were not significantly different. However, the downregulation of CyclinD1 expression was observed in overexpression of ALKBH5 compared with control (Fig. 5K, L). The data supported that METTL3 might play an important role in embryonic neural tube development by activating Wnt/β-catenin signaling. These suggested that ethionine could inhibit Wnt/β-catenin signaling pathway, and cause changes in the expression of downstream proteins and resulting in imbalance between cell proliferation and apoptosis, and participating in neural tube development.

### Knockdown of METTL3 and overexpression of ALKBH5 disrupt the balance of cell proliferation and apoptosis

**Fig. 6:**
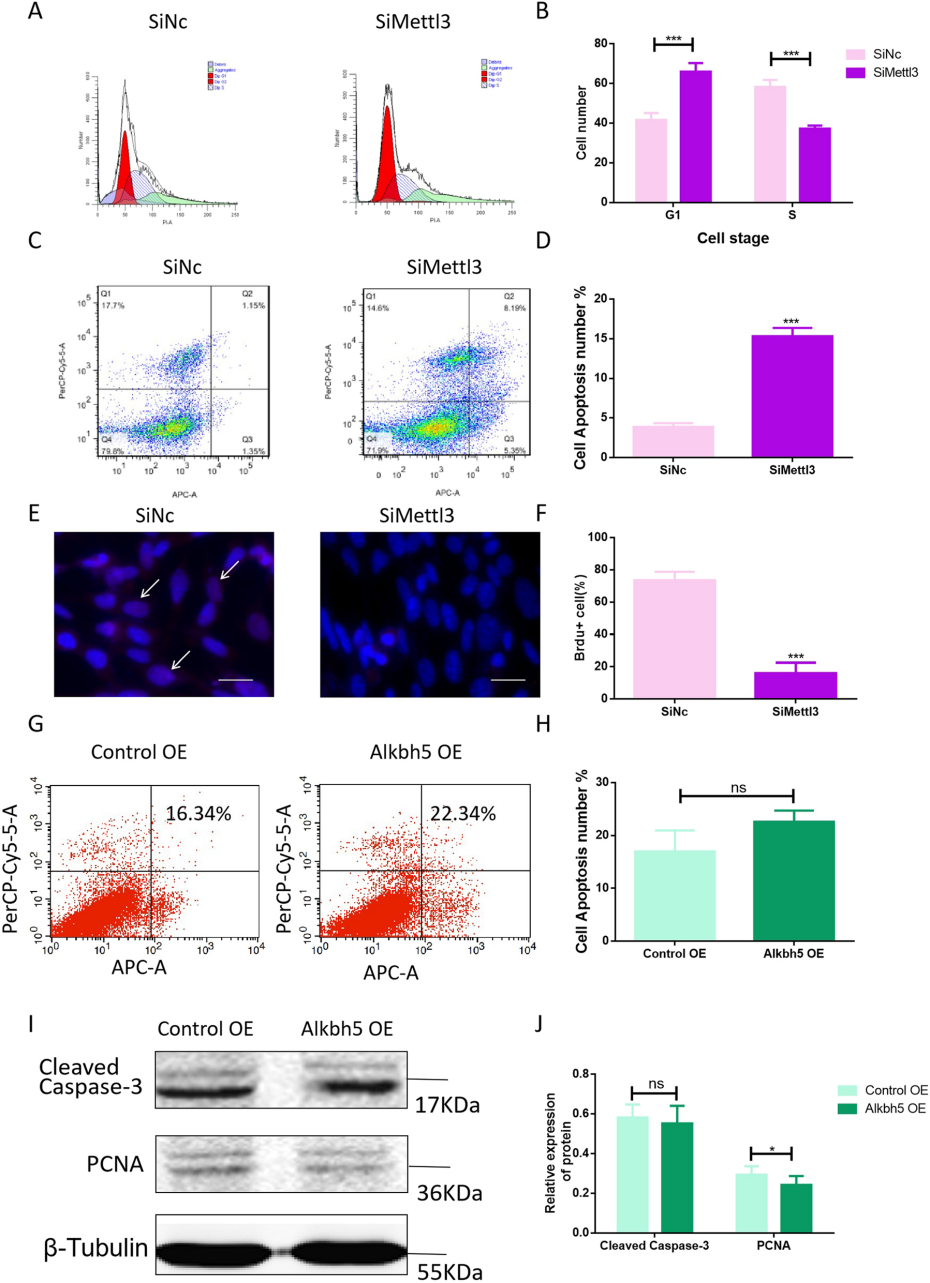
Knockdown of METTL3 and overexpression of ALKBH5 disrupt the balance of cell proliferation and apoptosis. **A**, **B** HT-22 cells were treated with wild type (SiNc) and METTL3 knock-out (SiMettl3), and cell cycle distribution was analyzed by flow cytometry. The percentages of cells in cell cycle phases were calculated from three independent experiments. **C**, **D** Apoptosis in HT-22 cells was measured and analyzed by flow cytometry. The combined early and late apoptosis rates were up-regulated significantly in the SiMettl3 group compared with those in the SiNc group. **E**, **F** The Brdu-positive cell of HT-22 cells from SiMettl3 was lower than that from SiNc.  ∗ ∗∗ *p* < 0.001 vs SiNc group. **G**, **H** Apoptosis in control OE and Alkbh5 OE HT-22 cells was measured and analyzed by flow cytometry. **I**, **J** Western blot analysis of the protein levels of total PCNA and Cleaved Caspase-3 in control OE and Alkbh5 OE HT-22 cells. Bar graphs for protein abundance were quantitative data from three independent experiments. β-Tubulin was used as a loading control

Next was to further examine the function of METTL3 and ALKBH5 in HT-22 cells, and elucidate the functional roles of m6A modification in NTDs. Above all, flow cytometric analysis was performed to analyze the biological role of METTL3 in cell cycle distribution. The cell cycle results showed that the population of cells in the G1 phase was significantly increased after knockdown of METTL3. In contrast, the cell quantity in the S phase was reduced in the SiMettl3 group compared with the SiNc group (Fig. 6A, B). Moreover, we examined the effect of knockdown of METTL3 on cell proliferation and apoptosis by Brdu and flow cytometry. It was also found that the SiMettl3 group cells showed high rate of early and late apoptosis compared with cells in the SiNc group (Fig. 6C, D). Additionally, Fig. 6E, F shows that the ratio of Brdu-positive cells was significantly decreased in SiMettl3 group compared with SiNc group, suggesting that the downregulation of METTL3 significantly promoted cell apoptosis formation and repressed cell proliferation.

To clarify the function of ALKBH5, we investigated the effect of overexpression of ALKBH5 on cell apoptosis in HT-22 cells by flow cytometric. Results found that there was no significant difference in Alkbh5 OE group and control OE group (Fig. 6G, H). At the same time, Western blot analysis was performed to detect Cleaved Caspase-3 and PCNA expression in HT-22 cells. We found that when ALKBH5 was overexpressed, the expression levels of Cleaved Caspase-3 were slightly changed, and it was not statistically different (Fig. 6I, J). Western blot results also showed that when ALKBH5 was overexpressed, the amount of PCNA was drop in Alkbh5 OE group compared to control OE group (Fig. 6I, J), suggesting that ALKBH5-mediated m6A demethylation inhibits cell proliferation. However, ALKBH5-mediated m6A demethylation has no effect on cell apoptosis. Collectively, all these data suggest METTL3 may have a potential role in embryonic neural tube development.

## Discussion

NTDs are the most common and severe human birth defects, mainly due to the failure of neural tube closure during embryonic development, leading to severe neurological consequences and even fatalities [8, 29]. The generation, proliferation, differentiation, and migration of neural cells and the resulting morphological changes are the cytological basis of neural tube development, and any abnormalities in this process can cause neural tube defects [30, 31]. Recent studies have revealed that m6A methylation is linked to embryonic development [32, 33]. Moreover, m6A modification plays an important role in facilitating early development via regulation of SAM availability [34]. METTL3 is firstly identified as component of the methyltransferase complex and it is a key factor for m6A modification, METTL3 has effects on cell division in mammals [35]. Increasing evidences indicate that m6A modification plays an important role in mammals, and that the METTL3 mutation is lethal to both mammalian and plant embryos [34]. Recent research found that ALKBH5-mediated m6A RNA modification plays a critical role in cardiac differentiation [32]. However, the role of m6A RNA modification in neural tube development remains unknown.

The overall objective of the current study was to understand the complicated effects of m6A on neural tube closure, which might be the foundation for resolving the pathogenesis of NTDs. SAM is a provider of methyl donors in the body [36]. Our study had confirmed that SAM not only played a compensatory role, but also led to m6A modification changes in neural tube development and regulation. Furthermore, data also indicated that METTL3 might induce apoptosis and inhibit proliferation by activating Wnt/β-catenin signaling pathway, which provided new clue for clinical researchers and work. Thus, our data demonstrated for the first time, to the best of our knowledge, that m6A RNA modification played a key role in neural tube development. With this purpose, we first examined the effects of ethionine pretreatment on pregnant mice resulting in reduced levels of SAM metabolism on embryonic neuroepithelial cell proliferation and apoptosis. It was found that after pretreatment with ethionine, TUNEL-positive cells increased significantly, whereas PCNA-positive cells, which are indicators of proliferation decreased. The activation of Caspase-3 is one of the most important events in the process of apoptosis, so the detection of Cleaved Caspase-3 is a common method in the study of apoptosis. Subsequently, we also extracted E10.5 embryonic brain tissues and tested the effects of ethionine on proliferation and apoptosis using Western blot. The same experimental results were found, and the results showed that ethionine can induce cells reduced proliferative capacity and excessive apoptosis. Simultaneously, results found that after the treatment with ethionine, the same over-apoptosis, proliferation inhibition occurred at HT-22 cells by flow cytometry, Western blot, EDU and other experimental methods.

The Wnt/β-catenin signaling pathway is one of the key pathways for many gene expressions that regulate cell proliferation, differentiation, and survival [37, 38]. A recent study reported that m6A modification of FZD10 mRNA contributes to PARP inhibitors resistance in cells via upregulation of Wnt/β-catenin pathway [39]. The previous research group underwent low-folate culture and performed expression profiling, and they also found that the Wnt/β-catenin signaling pathway is involved in folic acid metabolism [4]. In our study, we found that ethionine inhibits the activation of the Wnt/β-catenin signaling pathway, leading to changes in the expression of classic proteins and downstream target genes, which is reflected as the expression of upstream classical protein Axin-2 was up-regulated. On the contrary, the expression levels of β-catenin and TCF-4 protein were significantly decreased. These results were consistent with Li et al. [4].

Increasing evidences show that transcription factors and epigenetic modifications jointly regulate and act on gene transcription and participate in the development of diseases [40, 41]. Researches have reported that m6A modifications are involved in various biological processes, including cell fate determination, embryonic development, and cell cycle control [42, 43]. Some studies have used SiMettl3 to interfere with oocytes, and it was found that the reduction of METTL3 can inhibit the translation efficiency of mRNA and destroy the degradation of mRNA, further indicating that the reversible m6A modification has a role in mammalian oocyte maturation and preimplantation embryo development [19]. At present, there are few reports about m6A and NTDs. However, current literature reports showed that m6A is closely related to embryonic development. Geula et al. conducted an in vivo tracking study on knocked out of Mettl3 mouse embryos, and found that knocking out Mettl3 is embryonic lethal. Knocked out Mettl3 embryos began to appear with defects in E5.5–7.5 [44]. Wang et al. found that after conditionally knocking out Mettl3 in brain tissue, cerebellar granule cells apoptosis increased and cerebellar hypoplasia [45]. Based on this speculation, we believe that m6A modification affects cell apoptosis, which is one of the important mechanisms of neural tube defects. In this study, we found *Mettl3* deletion reduced m6A modification, as well as inhibited cell proliferation and promoted apoptosis via inhibiting Wnt/β-catenin signaling pathway. Based on the importance of the *Mettl3* gene, after we silenced *Mettl3* gene, we observed changes in the cell phenotype and found that the level of apoptosis increased and the level of proliferation decreased. These results indicate that the deletion of ethionine and *Mettl3* gene can lead to imbalance in apoptosis and proliferation, and participate in the development of NTDs. Interestingly, our study indicated that m6A demethylase ALKBH5 overexpression could curb cell proliferation by decreasing the protein expression of CyclinD1. However, ALKBH5 overexpression had no effect on cell apoptosis, which was inconsistent with a recent study [46]. We considered that this inconsistency was caused by the environment and cell lines. Moreover, it would be interesting to determine whether there exist different mechanisms involved in ALKBH5 in neural tube development, which are waiting further exploration.

## Conclusion

In summary, we reported a new idea for the correlation between m6A and neural tube development. Our results demonstrate that SAM not only played a compensatory role, but also led to m6A modification changes in neural tube development and regulation. We first found that ethionine affected m6A modification by reducing SAM metabolism. Secondly, we confirmed that changes of METTL3-mediated m6A RNA modification inhibited Wnt/β-catenin signaling pathway. Finally, we verified that target downstream genes of the Wnt/β-catenin signaling regulated cell biological changes, causing imbalances between cell proliferation and apoptosis. From the perspective of disease prevention to treatment, this research revealed the important role of SAM in the development of NTDs, providing a good theoretical basis for further research on folic acid deficiency reduced NTDs, and new ideas and clues for clinical researchers and clinical work.

## Data Availability

All the data generated or analyzed during this study are included in this article.

## Abbreviations

NTDs: Neural tube defects
SAM: *S*-Adenosylmethionine
SAH: *S*-Adenosylhomocysteine
METTL3: Methyltransferases methyltransferase-like 3
METTL14: Methyltransferase-like 14
FTO: Fat-mass and obesity-associated protein
ALKBH5: Alkylation repair homolog protein 5
TUNEL: Terminal deoxynucleotidyl transferase dUTP nick end labeling
DMEM: Dulbecco’s modified Eagle’s medium
